# Pediatric Primary Care Telemedicine: Perspectives from English- and Spanish-Speaking Medicaid Enrollees

**DOI:** 10.1089/tmr.2024.0028

**Published:** 2024-12-09

**Authors:** Anne R. Links, Eliana M. Perrin, Sarah Polk, Divya Konduru, Shafkat Meraj, Nakiya N. Showell, Suzanne M. Grieb, Helen Hughes

**Affiliations:** ^1^ Department of Medicine, Johns Hopkins School of Medicine, Baltimore, Maryland, USA.; ^2^ Department of Pediatrics, Johns Hopkins School of Medicine, Baltimore, Maryland, USA.; ^3^ Johns Hopkins School of Nursing, Baltimore, Maryland, USA.; ^4^ Johns Hopkins School of Public Health, Baltimore Maryland, USA.; ^5^ Centro SOL-Center for Salud/Health and Opportunity for Latinxs, Johns Hopkins University School of Medicine, Baltimore, Maryland, USA.; ^6^ Johns Hopkins University, Baltimore, Maryland, USA.; ^7^ Office of Telemedicine, Johns Hopkins University, Baltimore, Maryland, USA.

**Keywords:** telemedicine, pediatrics, Medicaid, health care utilization, patient perspectives, qualitative

## Abstract

**Objective:**

To qualitatively explore Medicaid-enrolled parents’ and young adult patients’ perspectives and experiences with telemedicine in pediatric primary care.

**Methods:**

We conducted semi-structured interviews with participants to explore their experiences with telemedicine. Participants consisted of English- and Spanish-speaking parents and young adults (18–21) who engaged in a telemedicine visit between March 15, 2021 and December 31, 2022 at two pediatric primary care clinics whose patients are predominantly insured by Medicaid. A qualitative descriptive design was used to develop a taxonomy. Frequencies were obtained to identify the most prevalent themes.

**Results:**

Twenty-six participants (22 parents, 4 young adults) were interviewed. Twelve (46%) participants were English-speaking and 14 (53%) were Spanish-speaking. Four domains were identified, each further classified into themes: pre-visit expectations (option for in-person visit, general anticipation, and specific worries), visit experience (general sentiment, technology, and quality of care), comfort (with overall process, privacy, and communication), and feelings about telemedicine (advantages, disadvantages, and loss of telemedicine). Although many participants had negative expectations of telemedicine prior to their appointment, a majority indicated positive experiences with visits and concerns about a possible future where telemedicine visits at home were no longer covered by their health insurance.

**Discussion:**

Most participants indicated positive experiences with telemedicine and perceived negative impact if access was removed. Findings related to perceived quality of care, advantages, and disadvantages suggest that patient preferences and individual circumstances should be taken into account when choosing visit modality in similar settings.

## Introduction

Telemedicine has the potential to increase access to health care services, reduce patient costs and travel barriers, and increase patient and provider satisfaction.^1–3^ The COVID-19 pandemic necessitated a rapid transition to telemedicine. Following the issuing of waivers by the Center for Medicare and Medicaid Services in March 2020, there was a 154% increase in telemedicine visits compared with the same time frame in 2019.^4^ The rapid transition to telemedicine offers a unique opportunity to learn about the perspectives of groups who may have the most to gain from this convenient and accessible care delivery modality.

Data from before and after March of 2020 indicate that patients insured through Medicaid, persons of color, low-income individuals, and people of Latinx ethnicity had lower rates of telemedicine visits compared with patients with private insurance and non-Hispanic White patients.^5,6^ A report on telemedicine use in North Carolina Medicaid recipients indicated that during the pandemic Black and Latinx patients had lower utilization of telemedicine than their non-Hispanic White counterparts.^7^ Despite these concerns, many studies evaluating perspectives on telemedicine involve patients of higher socioeconomic backgrounds and individuals who are predominantly non-Hispanic White and English-speaking.^8^ It is unknown whether prior findings of patient perceptions can be generalized to pediatric populations or minoritized groups who face barriers to both medical care in general and telehealth in particular. Furthermore, evidence supporting the use of telemedicine in pediatric care has focused on rural populations, specialty care, or mental health.^6,9–13^ Few studies have demonstrated how telemedicine can best be leveraged within pediatric primary care models, particularly serving parents with limited English proficiency or assessing the impact of telemedicine on family experiences among patients enrolled in Medicaid. The current study seeks to fill these gaps.

The purpose of this study was to assess the perspectives of English- and Spanish-speaking Medicaid-enrolled parents of children less than 18 years and young adult patients to inform and improve pediatric telemedicine practices and policy.

## Methods

A qualitative descriptive design was used to explore Medicaid-enrolled parent and young adult experiences with telemedicine visits. Procedures were approved by the Johns Hopkins School of Medicine Institutional Review Board (IRB00284439).

### Interviews

Participants were recruited from two academic-based primary care clinics in Baltimore, Maryland, at which most patients (>85%) are insured through Medicaid. Parents of children (<18 years) and young adult patients (18–21) were eligible if they had experienced a telemedicine visit at one of these clinics between March 15, 2020 and December 15, 2021. We purposely recruited the majority of participants to be parents of patients age 0–12 or patients age 18–21 given unique challenges in teen primary care telemedicine visits.

A semi-structured interview guide was developed by the research team and used across interviews (Supplementary Data S1). Questions about experiences with and perspectives on telemedicine visits were asked in an open format to avoid influencing participant responses. Interviews were conducted in English or Spanish according to participant preference by a bilingual study team member (Stephanie Wells). Participants gave oral consent prior to interviews, which were completed in person or over Zoom. Participant demographics were collected prior to beginning the semi-structured interview. Interviews lasted 16–37 min and were audio-recorded with participant permission.

### Data analysis

The audio recordings were transcribed *verbatim*. Spanish-language transcripts were initially translated into English by the online software Deepl and then examined by bilingual research assistants for quality and accuracy. Segments presented for publication were translated again by Johns Hopkins Language Access Services to corroborate initial translation.

A qualitative descriptive design was used to develop a taxonomy that reflected the data and limited theoretical interpretation.^14–16^ Three study team members (A.R.L., H.H., and Stephanie Wells) reviewed 30% of transcripts independently to identify themes in participant responses. To organize the data, codes and subcodes within themes were then grouped to develop the taxonomy. This taxonomy was established based on prevalent codes from the reviewed transcripts and established prior to final coding. Two coders (S.M. and D.K.) independently coded all transcripts at the statement level by identifying any statement that corresponded to a given code in the taxonomy. Emerging themes not included in the taxonomy were identified and examined for prevalence following final coding. Any disagreements were identified and discussed by these coders and resolved through consensus. Following the initial resolution of coding, a third party (A.R.L.) reviewed coding to ensure agreement with the representation of codes at the interview level, by identifying whether a parent or patient had endorsed a given code at least once during the interview.

Interview-level coding was obtained to identify observed frequently discussed thematic material across participants. Differences based on the language participants spoke were also observed to identify themes discussed more often in English-speaking versus Spanish-speaking groups.

## Results

Twenty-six interviews were conducted. Twelve (46%) were conducted in English and 14 (54%) were conducted in Spanish. Four participants (15%) were young adults and 22 (85%) were parents. All patients were Medicaid-enrolled. Full participant characteristics are in Table 1.

**Table 1.** Participant Characteristics^a^ (*N* = 26)

Characteristic	Total *N* (%), *N* = 26	Spanish-speaking *n* (%), *n* = 14	English-speaking *n* (%), *n* = 12
Ethnicity/race			
Latinx (any race)	16 (62)	14 (100)	2 (17)
Non-Hispanic White	1 (4)	0 (0)	1 (8)
Non-Hispanic Black	7 (27)	0 (0)	7 (58)
Non-Hispanic Asian	2 (8)	0 (0)	2 (17)
English fluency			
Not at all	4 (15)	4 (29)	0 (0)
Not very well	8 (31)	8 (57)	0 (0)
Well	2 (7)	1 (7)	1 (8)
Very well	12 (46)	1 (7)	11 (92)
Participant age (M [SD])	33.7 (9.5)	32.9 (6.2)	34.7 (12.6)
Parent education			
<high school	13 (50)	10 (71)	3 (25)
>high school	13 (50)	4 (29)	9 (75)
Patient age^b^			
<2	9 (35)	6 (42)	3 (25)
2–12	12 (46)	7 (50)	5 (42)
13–17	1 (4)	0 (0)	1 (8)
18–21	4 (15)	1 (7)	3 (25)

^a^ All participants insured by Medicaid.

^b^ Patient age <18: children of parents who were interviewed. Patient age 18–21: young adult patients who were interviewed.

**FIG. 1.**
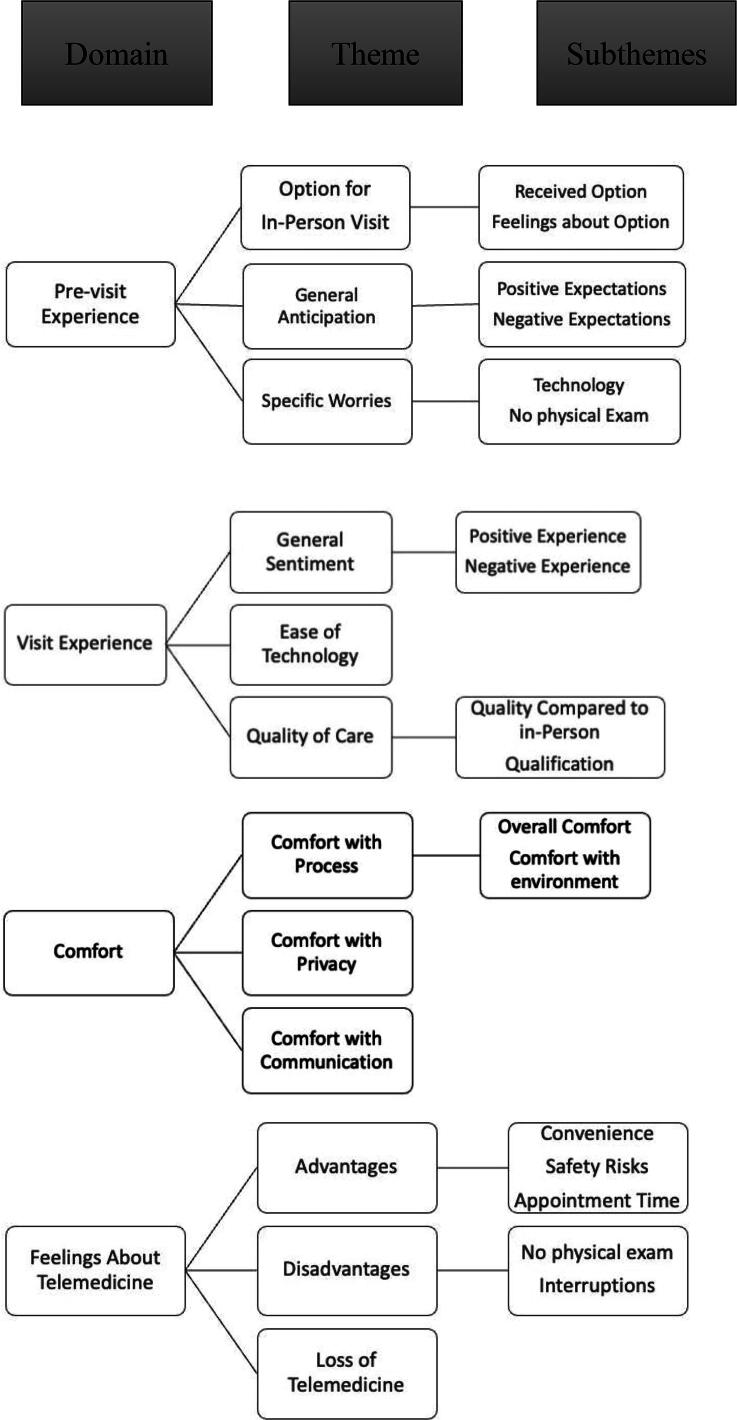
Taxonomy for parent and young adult patient experiences with telemedicine shows the domains and themes identified in parent interviews.

Four domains were identified; each further classified into themes: pre-visit experience (option for an in-person visit, general anticipation, and specific worries), visit experience (general sentiment, ease of technology, and quality of care), comfort (comfort of overall process, privacy, and communication), and feelings about telemedicine (advantages, disadvantages, and loss of telemedicine) (Fig. 1). Example quotes for each domain are provided in Tables 2–4.

**Table 2.** Examples of Quotation About Pre-Visit Expectations

Theme	*n* (%)	Example quotations
Pre-visit experience		
Option for in-person visit: Feelings about option	16 (62)	It made me feel a little more at ease with not having to-- I had that option of not coming in and having the possible exposure to other people and other patients coming in and out of the building, but it also made it easier as far as transportation (English-speaking parent)
General anticipation: positive expectations	7 (27)	*I was excited, especially with everything that was going on with COVID and everything, just knowing I was taking precautions to keep us healthy (English-speaking parent)*
General anticipation: negative expectations	14 (54)	*I didn’t like the idea much (Spanish-speaking patient)*
Specific worries: technology	10 (38)	*Just because I wasn’t real good with that computer setup I just was wondering how I was going to get it set up so I can see and make sure I get the appointment right (English-speaking parent)*
Specific worries: physical exam	8 (31)	*The doctor can’t diagnose something over video call (Spanish-speaking parent)*
Option for in-person visit: did not receive option	18 (69)	*They told me it could only be through video call (Spanish-speaking parent)*
Option for in-person visit: received option	9 (35)	*I was being offered the virtual and I opted for the in person (English-speaking parent)*

**Table 3.** Examples of Quotes About Visit Experience and Comfort

Theme	*n* (%)	Example quotations
Visit experience		
General sentiment: positive experience	21 (81)	It was very efficient; it worked out really well (English-speaking parent)
General sentiment: negative experience	11 (42)	*I don’t really like it much, and I think it’s better to go in person (Spanish-speaking parent)*
Ease of technology: easy	17 (65)	*It was easy (English-speaking patient)*
Ease of technology: difficult	8 (31)	*Since I’m not good with devices, I felt somewhat frustrated because I didn’t know how to log into those applications. It was a bit challenging for me (Spanish-speaking parent)*
Ease of technology: difficult but learned	7 (27)	*At first it was difficult, but then it became easier (Spanish-speaking parent)*
Quality of care: better compared with in person	2 (8)	*A little more with concern and comforting and being concerned about their patient to make sure that they touch bases on everything and they’re not forgetting anything or any concerns when they do virtual (English-speaking parent)*
Quality of care: the same compared with in person	12 (46)	*Everything is the same (Spanish-speaking parent)*
Quality of care: worse compared with in person	19 (73)	*I think in person visits are better (English-speaking parent)*
Quality of care: qualified	8 (31)	*It depends on what I was calling about, per se, if it was a rash or something that wasn’t really good being seen through video call. What else? Basically, it was okay for the most part, but it wasn’t the best thing at certain times (English-speaking parent)*
Comfort using telemedicine		
Comfort with process	10 (38)	*I’ve been feeling comfortable, and I haven’t had any issues (Spanish-speaking parent)*
Comfort with environment	5 (19)	*It’s good, because you’re in the comfort of your home (Spanish-speaking parent)*
Comfort with privacy (comfortable)	22 (85)	*They gave me instructions of the procedure and the HIPAA law and all that. They did good (English-speaking parent)*
Comfort with privacy (not comfortable)	4 (15)	*By video call you can make, I don’t know, a mistake or someone else might be watching you, and you get nervous (English-speaking parent)*
Comfort with communication (comfortable)	22 (85)	*I felt comfortable talking to her (Spanish-speaking parent)*
Comfort with communication (not comfortable)	9 (35)	*I feel like they might not understand what I want to explain (Spanish-speaking parent)*

**Table 4.** Examples of Quotes About Advantages and Disadvantages of Telemedicine

Theme	*n* (%)	Example quotations
Advantages		
Convenience, transportation	18 (69)	The advantages are that I don’t leave the house and... like I said, I don’t have transportation (English-speaking parent)
Convenience, parking	5 (19)	*You have to find parking, I park in a garage, or a lot, and time to get from there over to the whatever doctor’s office (English-speaking parent)*
Convenience: child care	4 (15)	*It was easy, because I didn’t have to leave my children somewhere else and have to pay (Spanish-speaking parent)*
Convenience: don’t miss school/work	3 (12)	*It was really convenient for me because I didn’t have to leave work early or take off. I just asked my boss if I could go on a later lunch break (English-speaking parent)*
Convenience: time savings	15 (58)	*I save a lot of time, and mostly, when I go in person, I have to kind of wait, but I just save all that like waiting (English-speaking patient)*
Safety risks	11 (42)	*It’s more safe than going to the hospital during the pandemic (English-speaking parent)*
Disadvantages		
No physical exam: condition requires an exam	19 (73)	*I had several video calls with her doctor about rashes or when some bump appeared, and obviously, a doctor can’t determine anything through a video call (Spanish-speaking parent)*
No physical exam: provide incorrect diagnosis	3 (12)	*The doctor couldn’t really diagnose, it was more like guessing because they prescribed allergy medication, and it wasn’t an allergy (Spanish-speaking parent)*
Interruptions	4 (15)	*I had to go somewhere the children wouldn’t be interrupting the call (Spanish-speaking parent)*
Future loss of telemedicine		
Negative feelings	17 (65)	*That would bring on a stress factor for me, big time, because like I said, with my work and my husband returning back to work not even two weeks ago, it makes life a whole lot easier for me knowing that I have that option to do virtual because I don’t always have someone to cover me at work (English-speaking parent)*
Neutral impact	10 (38)	*It wouldn’t affect me at all (Spanish-speaking parent)*

### Pre-visit experience

#### Option for in-person visit

Eighteen (69%) participants indicated that they were not offered the option of an in-person visit. However, 8 (31%) recalled having the option. One participant recalled having no option initially, but later being given the option of in-person visits. Some participants who had been offered an option expanded on their positive feelings about having a choice (“*It made it much easier having that as an option*”), while others who didn’t have the option of in-person care described feeling disappointed (“*I was a bit concerned because I had some things that I would have wanted to discuss in person*”).

#### General anticipation

Participants recalled their expectations of telemedicine when asked how they had felt about telemedicine before their visit, including positive (“*I felt good, because I don’t have to go to the clinic, exposing myself to getting infected with the virus, or infecting others*”) and negative (“*Nervous because I wasn’t sure how it was going to do it*”) feelings.

#### Specific worries

Participants indicated specific worries about telemedicine, such as technology (“*I worried I wouldn’t be able to connect*”), and not having a physical exam (“*How are they going to be able to diagnose anything that way?*”). Concerns about not having a physical exam included the exam in general (“*It’s not like being personally, in person, well, for them to look at the children. So, no, I didn’t feel good*”) and concerns about diagnosis or medication prescription (“*I wanted them to see her in person to see what she had or if they gave her any medicine*”).

### Visit experience

#### General sentiment

Participants expressed positive (“*it was good*”) and negative (“*I don’t believe in phone appointments*”) general views following their visit. Of note, nearly half of participants who initially had negative expectations indicated overall positive sentiment after the visit. For example, one parent who indicated in advance of their visit that they were “*nervous because I wasn’t sure how to do it*” after having a telemedicine visit said, “*it was very helpful.*” Another participant remembered questioning “*how am I going to do this?*” initially, but later appreciated the convenience of not having to travel (“*liked it because I wasn’t going anywhere*”). Reasons for positive telemedicine sentiment included convenience and reduced chance of infection. One parent said: “*It’s helped me a lot because I was afraid, afraid of going to the hospital and getting infected.*” Reasons for negative telemedicine sentiment often referenced lack of a physical exam: “*it’s different because, through video calls, they can’t touch the child, you only have to point to where it hurts, and the doctor has to draw conclusions.*”

#### Ease of technology

The majority of participants indicated technology was easy to use (“*I don’t have any problems*”), and others indicated that they learned how to use it (“*after they explained…it became easy for me*”). A minority had trouble with technology (“*I felt somewhat frustrated because I didn’t know how to log into those applications. It was a bit challenging for me*”). A few participants mentioned needing equipment, some of whom had the requisite resources readily available (“*Yeah, I had to use my own device, but I had no problem with that*”), although one participant stated the importance of having cell phone coverage with “*4G or higher*” and another indicated that their “*biggest challenge would be making sure the devices that we’re using are compatible.*”

#### Quality of care

Participants had varied perspectives on the quality of care. Many indicated that telemedicine was not as good as in-person visits (“*it’s much better in person*”) and that without a physical exam, their child’s condition could not be treated (“*it’s different, right? Because in a video call, it’s about questions and all that, and in person, they actually check your heart, lungs, and everything*”). While some spoke in broad terms (“*it’s not the same as seeing them in person*”)*,* others referenced specific conditions that required in-person visits (“*I had several video calls with her doctor about rashes or when some bump appeared, and obviously, a doctor can’t determine anything through a video call*.”). One parent emphasized:

The virtual one will never be the same as in person, right? Because, well, you can’t show the doctor where the child is hurt or where the problem is, right? To the doctor, I mean, then the doctors do the best they can, as much as they can, but, unfortunately, on a screen, well, no, you can’t do much, right?

Multiple participants who expressed negative perspectives of quality of care also expressed positive perspectives in the same interview.

Nearly half of participants considered telemedicine the same as in person (“*it was just like normal, the same as in person*”) or better (“*they went beyond*”). Some referenced clinicians’ ability to engage over video (“*I would compare that it’s about the same, if not a little more with concern and comforting and being concerned about their patient*”) or equal patient care (“*They made sure to ask me anything that they would ask me in person and give me the right care*”). One participant indicated providing clinicians medical measurement while using telemedicine, addressing concerns some participants had over lack of a physical exam:

I think I was all for the video visits, even down to like the pressure and everything. They ask ‘Do you have a cuff on hand? You could take the pressure. Do you have a scale on hand? You could take your weight.’ It was like things as if you was there. I could take the baby’s temperature, and they would explain to me where to take it at, or I can take my daughter’s temperature or take my son’s temperature.

Participants sometimes qualified quality of care, believing that telemedicine was effective in particular contexts (“*if I had something physically wrong with me, I would have needed her to see that in person, but other than that it was almost the same experience*”). Participants considered telemedicine to be a good option unless a physical exam was necessary: “*It’s convenient. I feel like if no wounds need to be looked at or it’s not an x-ray I think telegramming [sic] is awesome*.” One parent summed that: “*Sometimes it’s good and sometimes I felt as though I should have been able to come in for the appointment. It depends on what I was calling about, per se, if it was a rash or something that wasn’t really good being seen through video call.*” Participants who qualified their assessment of quality of care did so based on whether a physical exam was needed for a particular visit (“*my kids…need probably like hands-on stuff, because over the telecommunication it was okay, but the things the doctor couldn’t check them and touch them but can ask them all the questions*”).

### Comfort using telemedicine

Participants discussed comfort with telemedicine, including overall comfort, communicating with clinicians over video, and privacy (Table 3).

#### Comfort with the process

Many participants felt comfortable with telemedicine (“*She treated me very well, the questions, there were no issues. I felt really comfortable*”). Some felt comfortable with the process overall (“*I’m perfectly comfortable with it*”), while some felt comfortable because telemedicine occurred within their homes. Some participants spoke about primary care providers helping them to be comfortable with the process (“*It’s been pretty easy. Dr. X has made it super easy, and if I had any questions…I would also email MyChart and she would always answer within 24 h*”), or easing their initial concerns through the treatment process (“*at first it seems a little uncomfortable, but when you’re with them, they’re kind, they pay good attention to you, so you start to feel a little more comfortable*”).

Participants also felt comfortable due to experiencing telemedicine in their home environment (“*I feel comfortable as opposed to going to the clinic, well, it’s like they come to my house.*”). This was spoken of in terms of general comfort (“*it’s good because it’s in the comfort of your home*”) and feeling comfortable using their personal space (“*I feel comfortable because, I mean, as I’ve always said, my house is their house and…. I feel comfortable as opposed to going to the clinic, well, it’s like they come to my house.*”).

#### Comfort with privacy

A small number of participants indicated privacy concerns (“*when you’re in a room, you and your doctor, well, it’s more confidential, right?*”). The majority stressed that there had been no issues (“*No, the privacy was really good*”). One participant shared that privacy in telemedicine was identical to being in clinic (“*They ask that you be in a room where you have privacy for the appointment. If you’re going in person, you’re going to be put in a room where they respect your privacy as well.*”). Others shared the perception that privacy during telemedicine reflects in-person visits (“*it’s the same as if I would’ve been…there. It would’ve been me, the doctor, the walls…for the most part it was on the video me, the doctor, the walls*”). Another participant stressed the staff’s role in making them comfortable with privacy (“*They gave me instructions of the procedure and the HIPAA law and all that. They did good*”).

#### Comfort with communication

Participants indicated more positive than negative sentiments about communicating via telemedicine. Participants who indicated negative perspectives described general negative views (“*In person, I get to see the doctor…and it’s much better. I can talk to him; it’s better than over the phone. It’s always better to see the doctor in person*”), and specific concerns such as the time afforded in telemedicine (“*In a video call, well, you can’t take too much time*”). However, the majority of participants had notably positive perspectives (“*I felt comfortable talking to her*”), including communication being the same as in person (“*It was like a checkup visit, and you got to see the doctor in person*”), individual providers facilitating communication (“*I think my provider was making me comfortable*”), and attention to aspects overlapping with quality of care (“*They didn’t care any less just because we were not in person. They made sure to ask me anything that they would ask me in person and give me the right care*”).

### Feelings about telemedicine

Participants identified overall impacts of telemedicine, including advantages and disadvantages of telemedicine and the ways in which losing the option of telemedicine in the future would impact them (Table 4).

#### Advantages of telemedicine

##### Convenience

Convenience was overwhelmingly identified as the principal advantage of telemedicine. This existed across several areas, including transportation and parking, which are difficult in person (“*I have to change, then drive all the way over there, and find a parking spot. It’s so hard*.”), lack of childcare (“*I don’t have to struggle to take out both children*”), not having to miss work or school (“*it was really convenient for me because I didn’t have to leave work early or take off*”), and saving time (“*it makes it easier time-wise*”). Transportation was discussed both in terms of general convenience (“*you were able to just go downstairs to do the visit rather than having to get in a car, drive down there, find parking. I feel like it was more easier*”), and as a reason telemedicine allowed patients to seek medical care (“*If you don’t have a car, you have to call a taxi, take the taxi, and go. So, that’s the part that is a bit difficult often due to transportation. Sometimes you don’t have enough money for a taxi to go*”). One parent added: “*Yes, it’s different, because at home I don’t go out, I don’t pay for a taxi, I just get the child ready and that’s it. On the other hand, to go to the clinic, well, I have to call a taxi, or I have to walk and then I have to wait, so it’s different.*”

##### Safety risks

Participants identifying advantage of avoiding infection often referenced the pandemic, including general safety (“*it’s more comfortable or safer than coming to the hospital or clinic*”), COVID-specific references (“*It’s more safe than going to the hospital due to the pandemic*”), and overall concern: “*It’s not paranoid. It’s just a safety risk…you never know what’s on people’s mind, and you have to really take that very serious out here in this world today.*”

##### Appointment time

Participants also identified the length of telemedicine visits as an advantage, stating that the shorter appointments were both easier (“*it was just fast and easy and after she did one child she went to the next and then it was over*”) and more direct (“*get to the point and done and out*”). However, other participants identified these shorter appointments as a disadvantage, stating that the communication with the clinician was truncated and they felt rushed (“*In an in-person appointment you can talk more, you can have a chat with the doctor. In contrast, in a video call, well, you can’t take too much time*”). One parent summed that “*the disadvantages are that, sometimes in person, you receive it in depth, and in video they can’t do that.*”

#### Disadvantages of telemedicine

##### No physical exam

Overwhelmingly, participants discussed not having a physical exam as a disadvantage either consistently (“*I’d like to have the child checked out in person*”) or for particular visits (“*I had several video calls with her doctor about rashes or when some bump appeared, and obviously, a doctor can’t determine anything through a video call.*”). This concern was often raised because parents believed their child’s condition required a physical exam, including need to receive basic aspects of care such as obtaining vital signs or biological measurements (“*there could definitely be disadvantages if it was something that needed physical…where they check with the stethoscope*”) and needing their body examined: “*not being able to touch our child with their hands or see the child face-to-face, in person, it’s not the same, a video call and in person. So, that was not beneficial.*” A subset of participants also stressed the perspective that clinicians could not accurately diagnose medical conditions over video (“*Could she really tell if I’m really sick?*”) or prescribe the appropriate medical treatment (“*the issue is that they might not treat the child properly*”).

##### Other disadvantages

Disadvantages less often identified included being interrupted by family (“*I had to go somewhere where the children wouldn’t be interrupting the call.*”) and having shorter visits (“*there is less communication and fewer details*”).

#### Loss of telemedicine

Participants discussed the impact that a possible future loss of telemedicine would have (Table 4). The majority of participants (17, 65%) felt they would be impacted negatively if the option for telemedicine were removed, including being inconvenienced (“*I mean, I would be a little disappointed because it is, like I said, super convenient*”), miss the benefits of telemedicine (“*If I were sure that COVID is not coming back…this gets worse again, it will be difficult because then we will really need to have telemedicine*”) or seriously negatively affected (“*it shouldn’t go away, because if it does, then we’ll be left with nothing*”). One participant stressed, “*That would bring on a stress factor for me, big time, because… it makes life a whole lot easier for me knowing that I have that option to do virtual because I don’t always have someone to cover me at work.*” No participant indicated positive feelings about the prospect of telemedicine no longer being available. Participants who expressed neutral views sometimes did so because they currently utilized in-person visits (“*I wouldn’t have any issues because I like going there*”).

#### Differences in perspectives

Table 5 shows patterns stratified by language spoken. More Spanish-speaking than English-speaking participants expressed negative views of quality of care (“*the doctors do the best they can, as much as they can, but, unfortunately, on a screen, well, no, you can’t do much*”) and had difficulty using the required technology. However, when discussing specific disadvantages of telemedicine approximately half of Spanish-speaking participants mentioned the disadvantage of not having a physical exam because their child’s condition benefited from the exam, while nearly all English-speaking participants identified it as the primary disadvantage. However, the three participants who explicitly stated that clinicians cannot make a diagnosis or prescribe appropriate medication over telemedicine were Spanish-speaking (“*they might not give me the exact medication for the child because they can’t see him*”). Additionally, fewer Spanish-speaking participants identified aspects of convenience that some English-speaking participants emphasized (e.g., transportation, parking) as primary advantages of telemedicine. However, more Spanish-speaking participants discussed increased comfort due to conducting the visit from their home (“*it is more comfortable, you are at home, and you can answer patiently*”).

**Table 5.** Frequency of English- and Spanish-Speaking Responses to Primary Codes

Code	Spanish	English	Quotes
Pre-visit experience			
Positive expectations	3 (21)	4 (33)	**Spanish:** *I felt fine because I knew we couldn’t go to the hospital because of the virus.* **English:** *I was excited, especially with everything that was going on with COVID and everything.*
Negative expectations	10 (71)	4 (33)	**Spanish:** *it’s not like being in person, where they can see the children. So, no, I didn’t feel good* **English:** *I didn’t think it was going to be as efficient.*
Visit experience			
Positive experience	10 (71)	11 (92)	**Spanish:** *The in person appointment is more difficult.* **English:** *It was good. It helped a whole lot.*
Negative experience	8 (57)	3 (25)	**Spanish:** *I think it’s better to go in person.* **English:** *I don’t really like video visits.*
Worse quality of care	13 (93)	6 (50)	**Spanish:** *It’s not the same as going in person, having an examination, or checking the baby thoroughly right there.* **English:** *I think in-person visits are better. Just for the physical contact of it.*
Better/the same quality of care	3 (21)	11 (91)	**Spanish:** *Everything is the same* **English:** *it’s about the same, if not a little more with concern and comforting and being concerned about their patient to make sure that they touch base*
Comfort with telemedicine			
Felt comfortable with process	5 (36)	5 (42)	**Spanish:** *more comfortable, right? Because you can do it in your own home.* **English:** *I’m perfectly comfortable with it.*
Comfortable with privacy	11 (78)	11 (92)	**Spanish:** *Not [privacy concerns] at any time* **English:** *Yes. They was. They gave me instructions of the procedure and the HIPAA law and all that. They did good.*
Not comfortable for privacy	2 (14)	2 (17)	**Spanish:** *When you’re in a room, you and your doctor, well, it’s more confidential.* **English:** *We just have more privacy when we’re in the doctor’s office.*
Comfortable with communication	12 (86)	10 (83)	**Spanish:** *in terms of what we talked about with the doctor, that was very, very good.* **English:** *she took her time, she made sure to ask me if there’s any other issues that I’m experiencing before we got started.*
Not comfortable with communication	8 (57)	1 (8)	**Spanish:** *difficult part is that I feel they might not understand what I want to explain* **English:** *It’s easier to talk in person.*
Advantages, disadvantages, and loss of telemedicine			
Advantage of convenience	11 (78)	11 (92)	**Spanish:** *It’s easier.* **English:** *I think it’s really convenient.*
Advantage of safety risks	7 (50)	4 (33)	**Spanish:** *If you don’t go in person [you] avoid… getting infected, or being around other people* **English:** *It’s helped me a lot because I was afraid…of going to the hospital and getting infected*
Disadvantage of no physical exam	13 (93)	12 (100)	**Spanish:** *A doctor can’t determine anything through a video call.* **English:** *I’d like to have the child checked out in person.*
Negative feelings about Loss	7 (50)	10 (83)	**Spanish:** *Then we’lll be left with nothing…it should continue.* **English:** *that would be a stress factor for me.*
Neutral feelings about Loss	6 (43)	4 (33)	**Spanish:** *I wouldn’t have any issues because I like going there* **English:** *I wouldn’t feel any way. That’s fine.*

## Discussion

Participants seeking care at two Medicaid-focused pediatric primary care clinics had a positive overall view of telemedicine and were comfortable with the process. Mostly, participants did not think privacy was worse with telemedicine as compared with in-person visits. Their perspective on the quality of care of telemedicine was mixed, with most referencing lack of physical exam as a disadvantage. Participants overwhelmingly considered telemedicine more convenient than in-person visits, and a majority expressed negative feelings about the idea of losing telemedicine as an option in the future. This study fills an important gap in telemedicine research by exploring perspectives of a diverse, multi-lingual Medicaid-enrolled pediatric population.

Similar to prior studies, the overall perception of quality of care of telemedicine was mixed in this sample of Medicaid-enrolled parents and patients. In addition to variation of positive and negative views between participants, nearly half of the participants indicated both positive and negative feelings about quality of care in telemedicine at different points of their interviews. Research has found that the experience of care varies according to individual, social, and clinical complexity.^17^ Telemedicine has often been viewed positively across populations, however.^18^ A majority of participants in this study recalled telemedicine positively after completing at least one telemedicine visit. Half of the participants who had negative expectations about engaging in telemedicine reported positive experiences with their visits. This reflects previous research on patient perspectives of telemedicine; across contexts, clinicians and patients have responded positively to telemedicine.^19–22^ One study indicated that over half felt positive about the option, demonstrating a pattern of patients responding positively to telemedicine.^23^

Our results support the assertion that individual differences and preferences matter when determining if telemedicine is an appropriate care modality. For example, shorter appointments were both an advantage and disadvantage depending on the participant. This highlights the importance of patient choice in visit modality and is particularly pertinent considering that many of the participants in our sample indicated having no modality choice. Although the fact that a majority (69%) did not have the option for in-person visits is likely due to only one site having the capacity to see patients with COVID-like symptoms, a previous study indicated that approximately one third of participants were not offered a choice in modality, and one third did not receive their preferred modality.^24^ Participants’ frustration with not being offered a choice between telemedicine and in-person visits might be alleviated by an explanation for why telemedicine is appropriate with lack of necessity of a physical exam and exposure concerns, to aid in parent and patient understanding and confidence when an in-person visit is not available.

Our study describes perspectives of Medicaid-enrolled parents and young adults, who have been previously identified as being less likely to engage in telemedicine.^5^ Most prior studies have examined utilization patterns among patients enrolled in Medicaid but have not explored individual preferences and experiences.^25^ When asked openly to identify advantages, and disadvantages of telemedicine, participants echoed experiences, advantages, and disadvantages referenced in previously studied populations (e.g., convenience, lack of physical exam, transportation, COVID-19 exposure).^20,26^ Medicaid-enrolled participants had much in common with a qualitative study of mostly White, commercially insured parents, who indicated advantages of time, transportation, and reduction of infection, and disadvantages of lack of physical exam and technology difficulties.^8^ This suggests the possibility many benefits and pitfalls for telemedicine may be shared across populations.

Previous research has demonstrated disparities in telemedicine use based on race/ethnicity.^5^ In this study, a majority of Spanish-speaking participants indicated inferior perceptions of quality of care in telemedicine visits as opposed to only half of English-speaking participants, mirroring previous research indicating Latinx patients had less telemedicine use or tended to prefer in-person visits.^5,25,27,28^ Various explanations have been proposed to explain these differences, including access to resources (e.g., computers, internet) and language barriers.^5^ Participants in our sample echoed these concerns, including need for appropriate. Additionally, a greater proportion of Spanish-speaking participants found technology difficult to use compared with English-speaking participants. It is possible that Spanish-speaking patients require a greater degree of pre-visit assistance, contributing to disparities with satisfaction and technological ease, as the research team learned in an unpublished focus group with clinical primary care staff in Baltimore, Maryland (oral communication, September 2022). This provides a foundation to consider linguistic differences that may impact experience of telemedicine, to provide an accessible model of care, beneficial across populations.

Disadvantages perceived by Medicaid-enrolled participants largely reflected concerns shared by other populations, such as lack of physical examination and misdiagnosis.^8^ Lack of a physical exam was overwhelmingly considered a disadvantage of telemedicine. Previous research across age and socioeconomic groups supports this as a primary concern.^29^ There are virtual physical exam protocols that take many forms, including asking a child to jump when evaluating appendicitis or more systematic protocols such as the Patient-Assisted Virtual Physical Exam, a 10-step process where patients perform their own comprehensive physical exams under their doctor’s instruction.^30–32^ Depending on the results of this exam (e.g., swollen abdomen), a clinician may determine that an in-person visit is appropriate.^32^ Virtual exams show the potential to develop interventions to bridge patient concerns, but their disadvantages are amplified for populations who lack health literacy to consistently and accurately interpret medical problems when following their clinicians’ directives. Many Medicaid-enrolled participants found telemedicine to be beneficial unless the child had a presenting complaint that required an exam. This provides support for sustaining telemedicine as a standard option for appropriate complaints, introducing an equitable virtual physical exam, and directing children to in-person visits when appropriate.

Perceived advantages for telemedicine in the demographically diverse Medicaid-enrolled sample also mirrored the perspectives of White, commercially insured populations, including identified benefits of telemedicine such as decreased travel time.^8,20,26^ Overall, participants had one overwhelming reason for preferring telemedicine: convenience. This advantage has implications across populations but may be particularly notable in lower income populations who may lack resources such as transportation or the ability to take time off work.^33,34^ In some cases, the term convenience may underemphasize the importance of this care modality for patient access, as there are parents for whom telemedicine offers them the ability to receive health care that would otherwise be logistically difficult or even impossible. If the option for telemedicine were removed, this would have a definitive impact on parents who value telemedicine for easy access to medical care, such as allowing patients to balance appointments with their daily responsibilities.

There are a number of limitations to consider when interpreting the study findings. Medicaid-enrolled participants were recruited exclusively from two outpatient clinics in Baltimore, limiting the generalizability for environmental and clinical context. Because the sample of young adult patients was small (*n* = 4), we were unable to adequately explore differences between parent and patient experience. We included only one parent of a teenage child in our sample due to the concern that parents may be less involved in a telemedicine visit, with teenagers being responsible for the description of their symptoms during the visit. Future research is needed to better understand the use of telemedicine for teenagers, given the unique aspects of care for this age group. Additionally, our representation of parents’ and young adults’ experiences is based on parent recall of visits as opposed to their experiences at the moment. The accuracy of that recall may have varied based on the amount of time that had elapsed between their visit and their interview.

In summary, Medicaid-enrolled participants indicated positive views of telemedicine, although the evaluation of the quality of care compared with in-person visits was mixed. Telemedicine was considered positively due to convenience, but negatively when a physical exam was necessary. Many participants were concerned by the idea of telemedicine being removed as an option. Perspectives suggest that telemedicine is a positive option in pediatric primary care for Medicaid-enrolled patients whose condition does not require a physical exam, but that in-person visits should also be available when an exam is medically indicated. Responses also demonstrate the importance of patient choice in determining the benefit of telemedicine for the individual patient. State Medicaid policy should continue to cover telemedicine visits but also ensure patient choice for in-person visits when preferred.
