# Supplementary material for: Pediatric Primary Care Telemedicine: Perspectives from English- and Spanish-Speaking Medicaid Enrollees
Source: Telemed Rep. 2024 Dec 9;5(1):380–92. doi: 10.1089/tmr.2024.0028 (PMC11693964; doi:10.1089/tmr.2024.0028)
Supplement: Supplementary Data S1 [file tmr.2024.0028_supplementary_data.docx]

**Interview Questions: Patient/Family Perspectives on Telemedicine**

***If participant is the parent of a child < 18 who is a patient:***

As you know, the COVID pandemic changed how people received healthcare services in many ways. I would like to hear about your experiences seeing a doctor/clinician for your child through the phone or video, called telemedicine, during this time. We would like to hear about your experience with our clinic and other doctors who care for your child.

***If participant is a patient between ages 18-21 who is a patient:***

As you know, the COVID pandemic changed how people received healthcare services in many ways. I would like to hear about your experiences seeing a doctor through the phone or video, called telemedicine, during this time. We would like to hear about your experience with our clinic and other doctors who care for you.

1. During this time, how have your experiences receiving healthcare services (in person) for [you OR your child] been impacted?
2. What are your thoughts and feelings about telemedicine visits in general?
3. Roughly, about how many telemedicine visits have [you or your children] had since the start of the pandemic?

3a. Have these been mostly by phone or video?

3b. Were these visits with [you OR your child’s] regular doctor or did you see different doctors via telemedicine visits? If different, how do you feel about this?

3c. Have you had telemedicine visits with providers outside of [*Harriet Lane Clinic* or *Children’s Medical Practice*]? If so, how were these visits similar to visits with this clinic? How were they different?

1. When you first found out [you OR your child] were going to have a telemedicine appointment, instead of going to the clinic in person, how did you feel?
2. For most of your video or phone visits, did you have a choice to see the doctor in person if you wanted to?
3. What was your comfort with the technology needed for the visits?
4. Can you walk me through the process of getting ready for your first telemedicine visit?
5. What has been it been like for you to have telemedicine visits with a doctor at [*the Harriet Lane Clinic/Children’s Medical Practice*]?
6. Overall, how do you feel about seeing a doctor through a **video** visit?
7. Overall, how do you feel about seeing a doctor through a **phone** visit?
8. What do you think are the most important advantages or benefits to seeing a doctor by video or phone compared to in-person visits?
9. What do you think are the most important disadvantages, or things you dislike, about seeing a doctor by video or phone compared to in-person visits?
10. At any time, have you had issues related to having access to a device to have a telemedicine visit (e.g., smartphone, computer)? If so, please tell me about this.
11. At any time have you had any issues related to internet access to have the telemedicine visit? If so, please tell me about this.
12. Have you experienced any other barriers to receiving care through telemedicine?
13. Have you had any issues or concerns with privacy?
14. Before the pandemic, many insurances did not cover healthcare visits over video/phone when patients are in their homes. These visits have been covered by all health insurance companies during the pandemic and will be for at least the next 2 years, but we are unsure if they will continue to cover this service afterwards. **How would you feel if telemedicine went away?**

1. Is there anything you can recommend to improve getting healthcare by video or telephone?
2. Is there anything else you think it is important for us to know about your experiences or perceptions of telemedicine visits that we have not already discussed?
